# Biological Mechanisms of Cancer-Induced Depression

**DOI:** 10.3389/fpsyt.2018.00299

**Published:** 2018-07-10

**Authors:** Kimberly Young, Gurmit Singh

**Affiliations:** Department of Pathology and Molecular Medicine, McMaster University, Hamilton, ON, Canada

**Keywords:** cancer, depression, mood, inflammation, HPA axis, glutamate, system x^−^_c_

## Abstract

Patients with cancer are more likely to develop depression than the general population, which negatively impacts their quality of life and prognosis. In order to identify effective antidepressants catered toward cancer patients, the biology of depression in the context of cancer must be well-understood. Many theories have emerged postulating the mechanisms underlying the development of depressive disorder. Here, we review the role inflammation, a hyperactive hypothalamic-pituitary-adrenal (HPA) axis, and glutamate excitotoxicity may play in cancer-induced depression. Hopefully, novel therapeutics targeting these dysregulated pathways may be potent in ameliorating depressive symptoms in the cancer population.

## Cancer and depression

It seems rather intuitive that cancer patients will be negatively impacted by their diagnoses; however, one must be cautious to differentiate between a patient who is appropriately sad vs. one who is clinically depressed. As it happens, depression is the only psychological disorder that disproportionately affects cancer patients relative to the general population (1). The incidence rate for depression is two to three times higher in the cancer population compared to their healthy counterparts (2). It is estimated that a third of cancer patients will find the experience distressing and have a comorbid psychiatric disorder, with the risk of suicide rising to approximately 2.5 times that of the general population in the first year post-diagnosis (2). However, interpreting depression within the cancer population as purely a reactionary outcome ignores the biological mechanisms and processes that play a part in its onset, undermining the legitimacy and significance of depression in the context of cancer. It is interesting to note that Prasad et al. (3) found that 4.6% of men were diagnosed with a depressive disorder in the 2 years preceding a prostate cancer diagnosis. Similarly, a meta-analysis done by McGee et al. (4) reviewed seven prospective longitudinal studies in order to investigate the relationship between depression and cancer. They found evidence of a link between depressive symptoms and a later diagnosis of cancer, though this relationship did not reach statistical significance. Another study also identified depression's robust effect on the incidence of cancer, increasing the Hazard Ratio (HR) by 29% in addition to elevating the mortality HR by 34% (5). The temporality of these three studies do not support the idea that being diagnosed with cancer is a necessary antecedent to the development of depression. Though a causal link between depression and cancer cannot be concluded with certainty, this data suggests that the biological environment that fosters the progression of cancer could beget depression at the molecular level in spite of the patient being unaware of their disease status. The psychological impact of a concrete diagnosis could certainly exacerbate depressive symptoms later on. Further investigations into how the biology of cancer and the cancer environment may contribute to the development of depression may inform treatment options targeted for this unique subpopulation.

Cancer patients could highly benefit from effective therapies to manage their depressive symptoms. Patients battling cancer who have comorbid depression experience a poorer quality of life and are less likely to seek or comply with treatments, a potential contributing factor to the correlation between depression and mortality in the cancer population (6, 7). Similar effects in terms of mortality were found between studies that looked at depression preceding a cancer diagnosis and those that looked at depression after the patient was diagnosed with cancer (8). Cuijpers et al. (9) reported a relative risk of excess mortality in depressed cancer patients of 1.61 (95% *CI* = 1.56–1.90) and Lloyd-Williams et al. (10) identified depression as an independent predictor of elevated mortality in cancer. Cancer patients who exhibit depressive symptoms have a 26% higher mortality rate, while those who receive a clinical diagnosis of major depressive disorder experience a 39% greater mortality rate (1). The difference between those two statistics suggest that clinical depression may be more likely than the presentation of depressive symptoms to impact a patient's will to live; not surprisingly, this has negative implications for the desire to pursue or adhere to therapies and may increase suicide risk (8). It is important to consider this difference when making interpretations about studies, as the framework for how depression is defined may affect its ability to be detected. For example, different studies utilize different criteria for depression—while some only look at patients with a clinical diagnosis of depression, others consider patients that present with symptoms without an accompanying medical diagnosis (8). In this regard, it is important to note that patients with depressive symptoms that are below criteria threshold for a diagnosis of depression are often overlooked in studies; therefore, available statistics and analyses may not be wholly representative of the scope of problem that depression poses within the cancer population (8). For example, the prevalence of depression has been found to be as low as 2% and as high as 58% in cancer patients, depending on how depression was assessed (6, 11–13). Criteria for a diagnosis of major depression also include symptoms that may overlap with symptoms of cancer or cancer treatment, making it harder for studies to accurately identify depression in cancer patients (6).

Contributing to elevated mortality, there exists a higher suicide rate among patients diagnosed with cancer relative to the general population (14). The risk for suicide may even be underestimated, as deaths of patients who commit suicide may be misinterpreted as being a result of cancer or natural causes (15). The fact that patients with depression are less likely to have social support compared to patients without depression could be a contributing factor to negative feelings of isolation that drive patients to consider suicide (8, 16). Llorente et al. (15) also found that a quarter of the cancer population they sampled viewed cancer-induced pain as suicidal motivation. These statistics highlight the importance of proactive efforts to monitor mental health statuses of patients for the duration of cancer treatment, as early interventions to ameliorate depressive symptoms have been shown to lead to improved survival (3). Health care providers should also strive to be self-aware of subconscious biases or stereotypes that influence how they interact with and treat cancer patients with depression (3).

## Biological mechanisms of cancer-induced depression

A relationship between cancer and depression is strongly supported in the literature. Yet, an exact mechanism linking the unique properties of cancer biology to the onset of depression has not been clearly identified. Here, we review three of the prominent theories that have been proposed: the role of inflammatory mediators, an overactive HPA axis, and excess concentrations of the neurotransmitter glutamate.

### Inflammation

People with major depression have been found to have elevated levels of pro-inflammatory cytokines (1, 17). However, the locus of activation of inflammatory pathways has not yet been pinpointed (18). Cytokines are released both peripherally, by macrophages and lymphocytes, and centrally, by astrocytes and microglia (19). Psychological and psychosocial stressors are both capable of initiating inflammation and can do so centrally or peripherally (18, 20). Chronic stress is correlated with increases in C-reactive protein (CRP) and interleukin (IL)-6, among other inflammatory mediators, and seems to act largely through microglia (18). The activation of immune pathways in the periphery—perhaps as a result of an infection—begets decreased neurogenesis in the brain, specifically key areas for behavior and cognition, and neuroinflammation (18, 20). Any prolonged stress response can have negative consequences for the brain and peripheral organs (20). Regardless of location of origin, cross-talk between the central nervous system (CNS) and periphery plays a large role in the onset of depressive symptoms during inflammation (21). Pro-inflammatory cytokines are thought to promote abnormalities complicit in the pathophysiology of depression, including neurotransmitter metabolism, neural plasticity, and neuroendocrine function (2, 18). It is also of interest to note that the relationship between inflammation and depression may be bidirectional: while cytokines may promote depression, the reverse situation where depression upregulates inflammatory signaling could also be true (22).

Given the role of inflammation in depression, it is not hard to conceptualize how depression may manifest itself in the context of cancer. Mediators of the immune system can be detected in essentially all types of cancer. Even in the early stages of cancer progression, tumors produce various factors including cytokines, chemokines, growth factors, and transcription factors; this includes IL-6, CRP, and tumor necrosis factor (TNF)-α, to name a few (16, 22, 23). In turn, these tumor-derived factors can initiate an inflammatory response from the body that can have either anti-tumor or pro-tumor properties. Cytokines are also produced as a result of cell death incurred by cancer treatments such as chemotherapy or radiation, which recruits immune cells to the site of injury, as well as inducing the production of cytokines by neighboring cells and initiating various signaling pathways (2).

Characterizing key biomarkers that contribute to depression would enable clinicians to identify cancer patients who are at increased risk of developing depression and/or those for whom conventional therapies may not be as effective. For example, a study by Jehn et al. (6) found that patients with depression had a significantly higher plasma concentration of IL-6 relative to patients without depression. Produced by both immune and non-immune cells, like tumor cells and tumor-associated macrophages (TAMs), IL-6 is one of the key biomarkers of depression (24, 25). Maes et al. (26) noted increased serum IL-6 levels not only in depressed patients, but also patients with treatment resistant depression. As a biomarker, IL-6 has a proposed sensitivity of 79% and a specificity of 87% (22).

### Neurotransmitter metabolism

Monoamine neurotransmitters have long been known to have significant roles in mood regulation in the brain. Of the various monoamines, which include dopamine and norepinephrine, serotonin—also referred to as 5-hydroxytryptamine (5-HT)—has perhaps garnered the most attention, especially with the wide range of selective serotonin reuptake inhibitors (SSRIs) that have emerged as antidepressants. Tumor-derived or -initiated cytokines are able to dysregulate serotonin synthesis via their ability to activate the enzyme indoleamine 2,3 dioxygenase (IDO). Widely distributed in the brain, kidneys, lungs, and immune cells, IDO has been found to be overly expressed in a variety of different cancers (20, 21). IDO converts tryptophan, the primary amino acid precursor of serotonin, into kynurenine (KYN). The consequences of IDO activation are two-fold: (a) decreased levels of tryptophan, resulting in serotonin deprivation; and (b) activation of the KYN pathway, the process whereby KYN is converted into neurotoxic metabolites (18, 21).

In astrocytes, KYN is converted to kynurenic acid (KYNA) whereas it is preferentially converted into quinolinic acid (QUIN) in microglia (18, 21). QUIN is a potent *N*-methyl-D-aspartate (NMDA) receptor agonist, resulting in excess glutamate release, oxidative stress, and astrocyte apoptosis–all of which lead to neurodegeneration and neural excitotoxicity linked to depression (2, 18, 20, 21). Stress and inflammation are both capable of activating microglia, thought to be linked to the neurodegenerative pathway—perhaps via QUIN—proposed to contribute to decreases in brain volume observed in patients suffering from chronic depression (20). KYNA, on the other hand, has suggested neuroprotective properties given its ability to inhibit glutamate release and its antagonistic effect on the NMDA receptor (18, 20).

Moreau et al. (27) developed an animal model of depressive-like symptoms in mice by inducing chronic inflammation using *Bacillus Calmette-Guerin* (BCG), a chronic activator of IDO both in the brain and the lungs. In the immediate aftermath of inoculation, only sickness behaviors were noted; however, these were later replaced with depressive-like behaviors that were sustained for several weeks. This suggests an association between inflammation, IDO, and depression that could be at play in the cancer environment.

### Neural plasticity

Within the brain, cytokines including IL-6 and TNF-α are typically tasked with promoting neurogenesis and offering neural trophic support; however, overactive immune pathways seen in cancer are thought to lead to dysregulation of these processes. This leads to a reduction in neural growth, in addition to increases in oxidative stress and glutamate release (18). Altogether, these abnormalities result in excitotoxicity that disrupts the plasticity of neural networks (21).

An increase in glutamate release as a result of the action of cytokines is further coupled with a downregulation of glutamate transporters on glial cells, which means that the increased synaptic concentrations of glutamate are further exacerbated by reduced reuptake (18). Under physiological conditions, astrocytes work to regulate local glutamate concentrations; however, under conditions of prolonged glutamatergic activation, NMDA receptors are over stimulated and neural apoptosis occurs as a result (18, 20).

Furthermore, these cytokines may promote the release of reactive oxygen species that contribute to oxidative stress. Glial cells in various brain regions that are significant in mood regulation, like the medial prefrontal cortex (mPFC), may become damaged in these environments and a pathological brain morphology that contributes to the onset of depression may be established (18).

### Neuroendocrine function

The activity of the immune system in cancer can also be linked to the activation of the hypothalamic-pituitary-adrenal (HPA) axis (19, 20). Cytokines, like TNF-α, IFN-α, and IFN-γ, have been demonstrated to potently stimulate the HPA axis (19, 28).

### HPA axis

One of the theories surrounding the biology of depression that may be especially relevant in cancer is the role of the HPA axis. When various stressors threaten homeostasis, our bodies initiate a coordinated stress response from the immune, endocrine, and nervous systems to mediate the stimuli. The primary actors involved are hypothalamus, pituitary gland, and adrenal gland; therefore, they are collectively referred to as the hypothalamic-pituitary-adrenal (HPA) axis (29). It is thought that chronic activation of this network leads to dysregulation of the HPA axis, resulting in many negative consequences for the body and homoeostasis (1, 8). Hyperactivity of the HPA axis has been robustly proven to be a hallmark characteristic of major depressive disorder (17).

The activity of the HPA axis begins in the paraventricular nucleus (PVN) of the hypothalamus, specifically the parvocellular subdivision. Here, hypophysiotropic neurons are responsible for producing and releasing corticotropin-releasing hormone (CRH) and arginine vasopressin (AVP) in response to aversive stimuli. Within the median eminence of the hypothalamus, CRH is secreted into the hypophyseal portal system, a network of blood vessels that connect the hypothalamus with the anterior pituitary gland–the next structure that makes up the HPA axis. The anterior pituitary gland contains corticotrophs which express two subtypes of CRH receptors (CRHRs), CRHR1 and CRHR2, though CRH binds to CRHR1 with greater affinity relative to CRHR2. After CRH binds to its receptor, which is a G-protein coupled receptor (GPCR), adenylyl cyclase is activated and initiates a cyclic adenosine monophosphate (cAMP) pathway. Ultimately, the outcome of this cascade is the release of adrenocorticotropic hormone (ACTH) from the pituitary cotricotrophs into the systemic circulation (29, 30).

The receptor for ACTH is known as the melanocortin type 2 receptor (MC2R) and can be found in the adrenal cortex, specifically on parenchymal cells of the adrenocortical zona fasciculata. The binding of ACTH to its receptor initiates yet another cAMP pathway; this time, the end result is the production and secretion of glucocorticoids, among other steroid hormones including mineralocorticoids. The predominant glucocorticoid in humans is cortisol, which regulates a plethora of metabolic and immune processes. Glucocorticoids bind to glucocorticoid receptors (GRs), which are widely expressed both centrally and peripherally. In the inactive state, the GR resides in the cytoplasm as part of a multimeric complex of chaperon proteins, including several heat shock proteins (HSPs). The binding of glucocorticoids induces a conformational change into its active form, dissociating GR from its complex so that it may translocate to the nucleus. Once inside the nucleus, the GR may bind to glucocorticoid response elements (GREs) or transcription factors to regulate the expression of various target genes (29, 31).

The HPA axis is regulated by negative feedback, whereby glucocorticoids—released as a result of HPA axis activity—binding to their respective GRs subsequently shuts of the cycle (31, 32). Unlike the mineralocorticoid receptor (MR), which has a high affinity for corticosteroids, the GR has a low affinity for endogenous steroid hormones. As a result, during a stress response when concentrations of these substances are higher than their basal levels, the GR is thought to be more important than the MR for regulation (30). Negative feedback is thought to occur at both the hypothalamus, on CRH secretion, and the pituitary, on ACTH release (29, 30). The hippocampus has also been suggested as a possible regulatory site based on its density of GRs and observations that the stress response is muted following infusion of glucocorticoids into this region (29). Yet, although these regulatory mechanisms are in place, a consistently observed pathophysiology in patients with depressive disorder is hyperactivation of the HPA axis (18, 31). This aberrant behavior is thought to result from faulty feedback inhibition due to desensitized GRs and, therefore, reduced responsiveness to glucocorticoids (6, 18, 20). Pro-inflammatory cytokines—including those released from tumors—are thought to contribute to this desensitization by impairing the translocation and/or function of GRs (6). As a result, hypercortisolemia is seen in approximately half of the depressed population, rising to an 80% prevalence among those with severe depressive disorder (31). Prolonged exposure to high concentrations of glucocorticoids may have pathological effects on brain morphology; for example, Colla et al. (33) reported that depressed patients have decreased hippocampal volumes compared to healthy controls, while de Kloet et al. (34) discussed the decrease in dendritic branching resulting from chronic stress. Studies have shown antidepressants to be effective in upregulating GR expression and normalizing GR function, allowing the HPA to respond appropriately to negative feedback mechanisms (35, 36).

A frequently discussed side-effect of HPA axis dysregulation is abnormal circadian rhythms of cortisol release. This phenomenon can be attributed to an interaction between the HPA axis and the circadian clock system. Humans experience an increase in cortisol levels in the morning whilst a decrease is seen in the evening. However, chronic stress leads to a blunting of the typical evening decrease in glucocorticoids (37, 38). For example, Jehn et al. (24) found that patients with major depression had higher cortisol concentrations relative to non-depressed patients both in the morning, at 8:00 a.m. and in the evening, at 8:00 p.m. Concurrently, Alesci et al. (39) found increased IL-6 concentrations in the morning among patients with major depressive disorder whilst Miller et al. (18) reported a correlation between cortisol blunting and IL-6 levels in patients in the advanced stages of cancer.

Given that pro-inflammatory cytokines act to stimulate the HPA axis, it is not hard to recognize how increased inflammation in depression and/or cancer can induce hyperactivity (28, 40). Under normal conditions, glucocorticoids are known to be potent and robust anti-inflammatory agents: they limit both the production and effectiveness of cytokines, suppress the proliferation of T cells, and inhibit various immune pathways (19, 25, 41). However, despite a high concentration of endogenous glucocorticoids, a surge of pro-inflammatory immunological actors is characteristic of depression and is thought to be attributable to glucocorticoid resistance developed by the immune system (18, 20). In summary, a paradoxical co-existence of glucocorticoids and inflammatory agents—resulting from delicate and complex altered bilateral communication between the HPA axis and immune system—frequently exists in depression.

### Glutamate

Glutamate is widely acknowledged as the major excitatory neurotransmitter in the CNS, functioning antagonistically to γ-aminobutyric acid (GABA), the major inhibitory neurotransmitter (42). As the major actor in the brain, glutamate plays an integral role in key processes like learning and memory by inducing long-term potentiation (LTP) (43, 44). LTP and its counterpart long-term depression (LTD) are two mechanisms of prolonged synaptic plasticity, strengthening and weakening the excitatory synapse, respectively (45).

The glutamatergic synapse is often referred to as the tripartite synapse due to the existence of three structural components: (1) a presynaptic neuron, (2) a postsynaptic neuron, and (3) glia (43). Altogether, these structures work in concert to achieve glutamate release, uptake, and clearance of glutamate. Within the CNS, glutamate can be produced using one of two processes: (1) *de novo* synthesis using glucose as a precursor or (2) via the glutamate-glutamine cycle (43, 46). In the latter pathway, glutamate in a synapse is taken up by astrocytes wherein glutamine synthetase converts it to glutamine; glutamine is then released and taken up by neurons that convert it back to glutamate using the enzyme glutaminase (46, 47). In the presynaptic neuron, glutamate is then packed into vesicles, a process aided by vesicular glutamate transporters (VGLUTs). Following depolarization of the presynaptic neuron and subsequent calcium influx, soluble N-ethylmaleimide-sensitive factor attachment receptor (SNARE) complexes aid in the exocytosis of these vesicles so that glutamate may be released into the synapse (43). After glutamate reaches the synapse, it is free to bind to its various receptors, which can be classified into two broad categories: the ionotropic glutamate receptors and the metabotropic glutamate receptors.

Once glutamate has been released into the extracellular space, regulation and clearance become highly important so as to avoid overabundant concentrations that impair both synaptic and extrasynaptic processes. Glutamate transporters found on all three elements of the tripartite synapse help in this process, specifically the excitatory amino-acid transporters (EAATs). To date, five EAATs have been characterized–EAAT1 through EAAT5. EAAT1 is primarily found on oligodendrocytes; EAAT2 is localized to astrocytes; EAAT3, EAAT4, and EAAT5 are mainly found on neurons, with EAAT5 being specific to retina (42, 46–48). As previously described, glutamate taken up by EAATs on astrocytes can then be funneled into the glutamate-glutamine cycle.

By this point, it should be evident that the regulation and coordination of glutamate release and clearance is tightly regulated. Yet, in depression—and a variety of other CNS disorders, neurodegenerative disorders, and neuropsychiatric disorders—glutamate has been measured in high concentrations in the brain, plasma, and cerebrospinal fluid (CSF) (44, 48). This leads to the excessive glutamatergic signaling that has become recognized as a hallmark of depression (2). These conditions result in excitotoxicity, which describes cell/neuronal death as a result of sustained activation beyond regular levels (49). Recall from the previous section detailing the HPA axis that a robust relationship between chronic stress and depression has been observed. Many animal models studying the link between chronic stress and depression have noted elevated extracellular glutamate levels in stress-induced depressed animals due to impaired clearance by EAATs and hampering of the glutamate-glutamine cycle (44, 48). Numerous studies of glutamate in mood disorders have utilized proton magnetic resonance spectroscopy (^1^H-MRS) to measure Glx which, put simply, reflects total glutamatergic availability, encompassing both glutamate and glutamine. ^1^H-MRS studies have found decreased Glx levels in the mPFC in patients with depression, coupled with increased Glx levels in the occipital cortex (50–52). One may infer a correlation between those findings and others that note neuronal atrophy and dysfunctional synaptic plasticity in the same brain regions (49, 53). In summary, excessive glutamate and glutamatergic signaling may induce neuronal degeneration and interfere with synaptogenesis, resulting in important cognitive and behavioral impairments.

It is also possible that peripheral tumors may play a role in elevated glutamate levels in the brain. Recall that tumors release various pro-inflammatory cytokines. Byproducts of inflammation are capable of activating matrix metalloproteases (MMPs) which, in turn, disrupt the basement membrane and tight junction proteins of the blood-brain barrier (BBB) (54–57). Other pathologies like chronic stress have also been implicated in disrupting the BBB. The BBB is tasked with regulating solute transport between the blood and brain, maintaining CNS homeostasis (58). When the integrity of the BBB is impaired, solutes are able to move with greater ease from the periphery to the brain. For example, lipopolysaccharide (LPS)-activated peripheral inflammation has been shown to lead to elevated cytokine levels and inflammation in the brain (59–60). Therefore, a disrupted BBB could be a route through which peripherally released glutamate from tumors can directly cause aberrant signaling centrally, in addition to acting from the periphery via signal transduction. This exact phenomenon has been demonstrated both in animal models and clinical research involving pathological states, including neurodegenerative disorders and gliomas (65).

Much attention has been focused on the NMDA glutamate receptor, specifically, when investigating the role of glutamate in excitotoxicity. After Olney (66) first observed the deleterious effects of glutamate on CNS neurons, many investigations were done looking into glutamatergic signaling and neuronal death. Rothman and Olney (67) later suspected the role of Ca^2+^ influx in glutamate-mediated neurotoxicity. Around the same time, others were already demonstrating that Ca^2+^ influx via the NMDA receptor played a key role in excitotoxicity (68–71). Interestingly, it is also widely known that neuronal development and survival are reliant on the activity of NMDA receptors (72, 73). It has long been suspected that the switch between beneficial and harmful glutamatergic activity happens upon escalation of NMDA receptor activity from moderate to excessive (73, 74). However, as of late, new theories have emerged into how the NMDA receptor may be implicated in excitotoxicity. Contrary to previous suppositions that these receptors were largely immobile, especially relative to AMPA receptors, the NMDA receptor has been found to be capable of lateral movement from synaptic to extrasynaptic sites (75). It is thought that NMDA receptors located at the synapse proper are linked to neuroprotective pathways, conferring neuronal survival, whereas extrasynaptic NMDA receptors trigger apoptotic pathways (73). Ca^2+^ entry via synaptic NMDA receptors potently activates cAMP response element binding protein (CREB), a transcription factor that is widely touted for its involvement in neuronal plasticity and overall survival (76–79). CREB then increases the expression of the *Bdnf* gene, which encodes the neuroprotective brain-derived neurotrophic factor (BDNF) (73, 76). BDNF promotes synaptic plasticity and, as the name suggests, neuronal growth (53). However, the same degree of Ca^2+^ influx that confers neuroprotection via synaptic NMDA receptors induces cellular damage through extrasynaptic NMDA receptor activity; this would seem to support the theory that it is the location of the receptor with reference to the postsynaptic density—and not the absolute amount of Ca^2+^ influx—that distinguishes excitotoxic signaling from regular signaling (76).

Notions surrounding the role of the NMDA receptor in excitotoxicity were further supported after ketamine, an NMDA receptor antagonist, garnered widespread attention as a fast-acting and long-lasting antidepressant (80–89). Ketamine, or (*RS*)-2-(2-chlorophenyl)-2-(methylamino)cyclohexanone, originally emerged in the 1960s as an anesthetic (82). It is characterized as a non-competitive, non-selective, and high-affinity antagonist of the NMDA receptor (83). Although its rapid effect makes it an attractive candidate as an antidepressant, especially when compared to the slow-acting monoamine-targeting pharmacotherapies (e.g., SSRIs), it does come with some unfavorable side effects. Even at subanesthetic doses, patients have experienced dissociative symptoms, altered perception, cognitive impairment, and even schizophrenia-like behaviors (90, 91). Yet, it is still a widely popular and efficacious antidepressant, ameliorating many depressive symptoms such as anhedonia and suicidal ideation (49). Given the success of ketamine as an antidepressant agent, glutamate has garnered a lot of interest as a potential therapeutic target for major depressive disorder.

### System ${\text{x}}_{\text{c}}^{-}$

Beyond vesicular release of glutamate from presynaptic neurons, there are also non-vesicular mechanisms of glutamate release; one in particular that has attracted a lot of interest is the system ${\text{x}}_{\text{c}}^{-}$ antiporter. A member of the heteromeric amino acid transporter (HAT) family, system ${\text{x}}_{\text{c}}^{-}$ is comprised of a heavy chain, 4F2hc, and a light chain, xCT, linked by a disulfide bond (92). xCT is credited with allowing system ${\text{x}}_{\text{c}}^{-}$ to transport amino acids and is therefore known as the functional subunit. 4F2hc supports local trafficking and is required for expression of the transporter on the cell surface (93). The system ${\text{x}}_{\text{c}}^{-}$ antiporter's structure has been studied and it is predicted to contain 12 transmembrane domains, intracellular N- and C-termini, and a re-entrant loop between loops 2 and 3. It is chloride-dependent, sodium-independent, and electroneutral (92).

**Figure 1:**
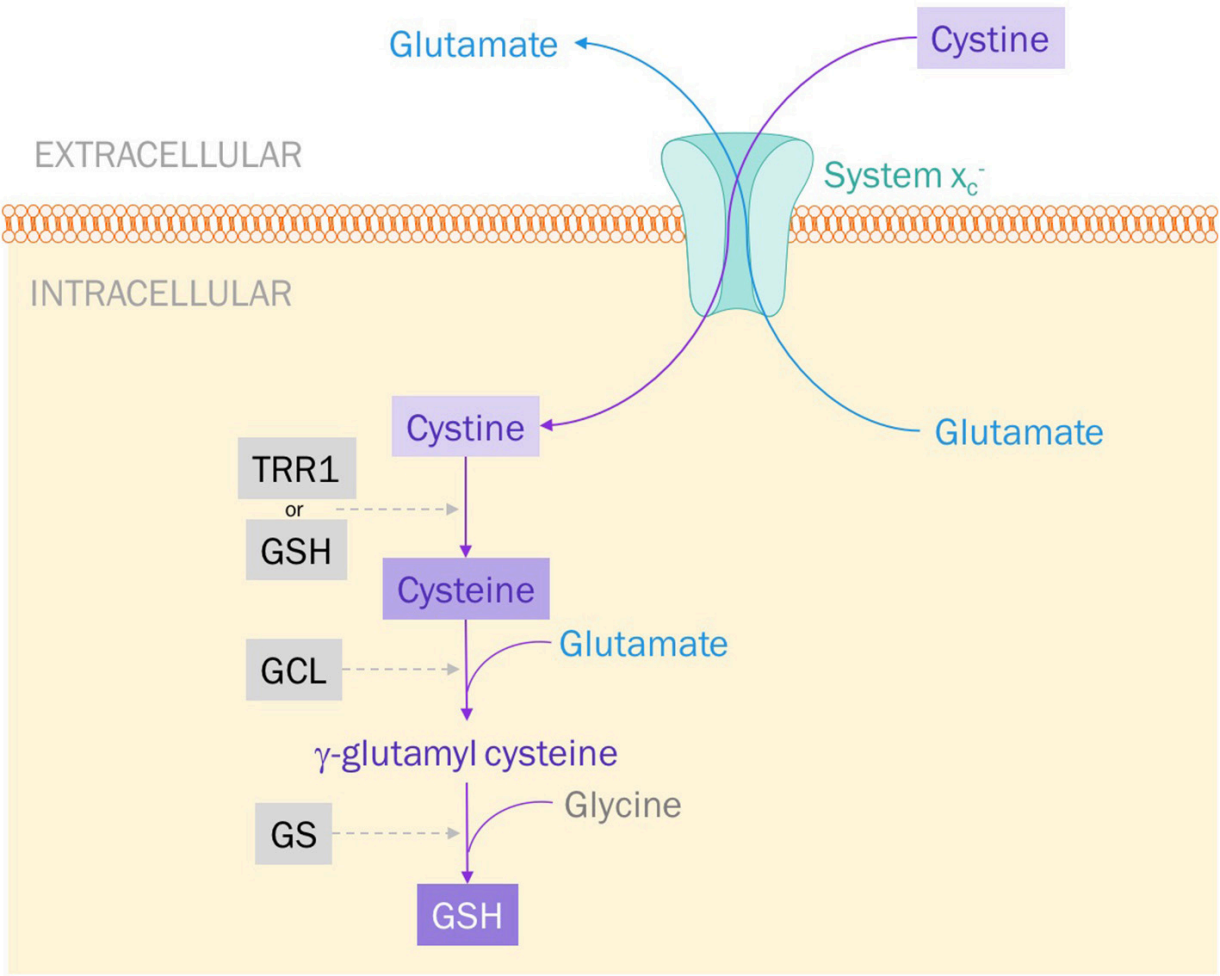
System ${\text{x}}_{\text{c}}^{-}$ exports glutamate and imports cystine. Cystine is reduced to cysteine by either thioredoxin reductase 1 (TRR1) or glutathione (GSH). Glutamate cysteine ligase (GCL) catalyzes the addition of glutamate to cysteine to produce γ-glutamyl cysteine. Glutathione synthase (GS) catalyzes the final step: the addition of glycine to produce GSH.

System ${\text{x}}_{\text{c}}^{-}$ is classified as an antiporter because of its bilateral exchange of amino acids; namely, intracellular L-glutamate for extracellular L-cystine at a 1:1 ratio (Figure 1) (94, 95). Though the antiporter can move either amino acid in either direction, it essentially exclusively imports cystine and exports glutamate (93). This is dictated by the concentration gradients of both cystine and glutamate established across the membrane: intracellular concentrations of cystine are negligible whilst extracellular glutamate levels are lower than intracellular levels (94).

System ${\text{x}}_{\text{c}}^{-}$ is the primary source for intracellular cystine and, therefore, plays a large role in protecting cells against oxidative stress. When a cell experiences high levels of metabolic activity, it produces free radicals—usually reactive oxygen species (ROS). It is the job of antioxidants to protect our cells—specifically, DNA and proteins-from the harmful effects of ROS. Glutathione (GSH) is one such small-molecule antioxidant. A tripeptide composed of glutamate, glycine, and cysteine, the synthesis of GSH is largely dependent on the intracellular availability of cysteine. Cystine imported via system ${\text{x}}_{\text{c}}^{-}$ is rapidly reduced to cysteine and incorporated into GSH production (Figure 1) (92, 96). This process is what keeps cystine at such low concentrations intracellularly. Inhibiting system ${\text{x}}_{\text{c}}^{-}$, therefore, could be a way to induce oxidative stress and apoptosis in cancer cells, making them more vulnerable to radiation therapy and other anti-cancer pharmacological agents.

Glutamate released from system ${\text{x}}_{\text{c}}^{-}$ can go on to participate in glutamatergic signaling. However, under pathological conditions, system ${\text{x}}_{\text{c}}^{-}$ may act as another route through which excessive amounts of glutamate may be released into the extracellular environment. Ye and Sontheimer (97) found that human glioma cells secrete excessive amounts of glutamate. It has since been observed that glioma cells highly express the system ${\text{x}}_{\text{c}}^{-}$ antiporter, using it for 50% of their glutamate transport across the cell membrane (98–100). Exacerbating the harmful effects of system ${\text{x}}_{\text{c}}^{-}$ upregulation in gliomas, EAAT2 expression has been found to be concurrently downregulated meaning that the excessive release of glutamate is not being cleared appropriately (101). However, system ${\text{x}}_{\text{c}}^{-}$ is not expressed exclusively by neuronal cells; in fact, its expression has been noted in immune tissues, the spleen, hepatocytes, and fibroblasts and a variety of cancer cell lines (92). Further, besides excitotoxicity, glutamate released via system ${\text{x}}_{\text{c}}^{-}$ may also elicit another phenomenon known as oxidative glutamate toxicity. As a competitive inhibitor of system ${\text{x}}_{\text{c}}^{-}$, glutamate can inhibit the import of cystine and interrupt GSH synthesis, depleting GSH supply in the cell and inducing oxidative stress (92).

## Conclusion

There are numerous mechanisms that are proposed to play a role in the onset of depression; inflammation, the HPA axis, and glutamate excitotoxicity were described. Currently, a paucity of studies assessing the effectiveness of antidepressants and non-pharmacologic options in cancer patients has resulted in the widespread use of traditional antidepressants, like SSRIs, as first-line treatment for cancer-induced depression. Insight into how the cancer environment may serve as an impetus for these pathophysiological phenomena could not only inform new antidepressant therapies targeted for this specific subpopulation, but also serve to identify cancer patients who may be at risk of developing depression during their battle with cancer. Alleviating depressive symptoms and the negative consequences of depressive disorder in the cancer population could improve patients' quality of life and prognoses.

## Author contributions

All authors participated in designing the concept of this manuscript. KY reviewed the literature and drafted the article. GS finalized the paper and provided suggestions to improve it.

### Conflict of interest statement

The authors declare that the research was conducted in the absence of any commercial or financial relationships that could be construed as a potential conflict of interest.
